# Case Report: Medical management of a rare infectious basilar artery aneurysm

**DOI:** 10.3389/fmed.2026.1872023

**Published:** 2026-06-04

**Authors:** Jie Wang, Yuhua Lv, Lihao Liu

**Affiliations:** Banan Hospital of Chongqing Medical University, Chongqing, China

**Keywords:** case report, cerebral infarction, infectious intracranial aneurysm, infective endocarditis, medical management

## Abstract

**Introduction:**

Ruptured infectious basilar artery aneurysms, which are complicated by hemorrhage, typically necessitate interventional therapy and are associated with an unfavorable prognosis. In contrast, successful outcomes following the administration of conservative medical therapy for unruptured infectious basilar artery aneurysms have rarely been reported.

**Clinical presentation:**

A 30-year-old male presented with acute confusion and dysarthria, which led to a diagnosis of cerebral infarction. Evaluation revealed a ventricular septal defect with secondary infective endocarditis (IE), which caused inflammatory embolization. After aggressive anti-infective therapy, his symptoms resolved. Subsequent vascular imaging demonstrated multiple basilar artery aneurysms. The family declined both interventional and cardiac surgical treatments, and the patient was maintained on antiplatelet therapy. Eight-month follow-up imaging revealed complete aneurysm resolution without rupture.

**Conclusion:**

Despite the rarity of infectious aneurysms, cerebrovascular imaging is recommended for patients with systemic infection and neurological symptoms. This case confirms that management strategies must be individualized, as unruptured infectious aneurysms can achieve complete resolution with pharmacological therapy.

## 1 Introduction

Infectious intracranial aneurysms (IIAs) are uncommon cerebrovascular complications of systemic infections, accounting for approximately 0.5–6.5% of all intracranial aneurysms (1). Infective endocarditis (IE) is the most common predisposing factor and is reported in approximately 69.8% of cases (1–4). Anatomically, IIAs occur most frequently in the anterior circulation (79.2%), with the middle cerebral artery being the most commonly affected vessel (56.9%) (1). Only a minority of cases (24.2%) involve the proximal branches of the internal carotid artery system, while basilar artery involvement is exceptionally rare (5).

Clinically, compared with noninfectious aneurysms, IIAs have a poorer prognosis because of a greater risk of rupture. The mortality rate following conservative medical management can reach 26.7% (1). Endovascular therapy is widely considered the first-line treatment for infectious intracranial aneurysms (IIAs) (6). However, according to the investigation by Rice et al., only 28% of patients experienced regression or complete resolution of IIAs following anti-infective treatment alone, despite its median duration of 36 days (7). This report presents an uncommon clinical manifestation of infective endocarditis, which was accompanied by cerebral infarction and an infectious basilar artery aneurysm. Under conservative medical management, the structural integrity of the aneurysm was maintained throughout the surveillance period, with progressive morphological alterations observed on serial imaging and complete resolution ultimately achieved. In addition, a comprehensive review of the relevant literature is presented.

## 2 Clinical presentation

A 30-year-old male presented to the emergency department with acute dysarthria and mental drowsiness. Physical examination revealed fever (38.3 °C) and a grade III holosystolic murmur with a palpable thrill at the left 3rd–4th intercostal spaces. The patient had no significant past medical or family history. Neurological examination revealed drowsiness, impaired right eye adduction and left eye abduction, and mild tetraparesis. Emergency diffusion-weighted imaging (DWI) revealed acute infarctions in the brainstem and left cerebellar hemisphere (Figures 1A,B), whereas computed tomography angiography (CTA) was unremarkable (Figure 1C). Laboratory findings included leukocytosis (11.9 × 10^9^/L), with 90.5% neutrophils, and a positive blood culture for *Streptococcus viridans*, whereas cerebrospinal fluid analysis revealed pleocytosis (493.00 × 10^6^/L) with an elevated protein concentration (44.50 mg/dL). Echocardiography confirmed a ventricular septal defect with mitral valve vegetation (Figures 2A,B), whereas chest CT excluded pulmonary infection. The cerebral infarction was attributed to embolization of the cardiac vegetation to the vertebrobasilar circulation. The patient received meropenem and vancomycin with low-molecular-weight heparin (4,000 units every 12 h). His consciousness normalized, although residual dysarthria and mild right-sided hemiparesis persisted. Follow-up magnetic resonance angiography (MRA) at 10 days revealed multiple basilar artery aneurysms and left posterior cerebral artery P1 segment stenosis (Figures 3A,B). The final diagnoses were (1) cerebral infarction, (2) IBA, (3) posterior cerebral artery stenosis, (4) IE, (5) ventricular septal defect, and (6) sepsis. The family declined interventional and cardiac surgical treatments. At discharge, heparin was replaced with aspirin (100 mg daily). MRA at 60 days revealed enlargement of the left basilar artery aneurysm, resolution of the right-sided aneurysm, and persistent P1 stenosis (Figure 3C). Remarkably, MRA at 278 days demonstrated complete resolution of all the vascular pathologies (Figure 3D). The final assessment revealed only mild residual dysarthria and occasional dizziness, with preserved motor function and independent living capacity. The patient’s wife stated that, due to financial constraints, they did not adhere to the physician’s recommendations for serial vascular assessments and timely surgical intervention for the aneurysm. She expressed surprise and satisfaction that the patient ultimately achieved a favorable prognosis through medical treatment alone.

**Figure 1:**
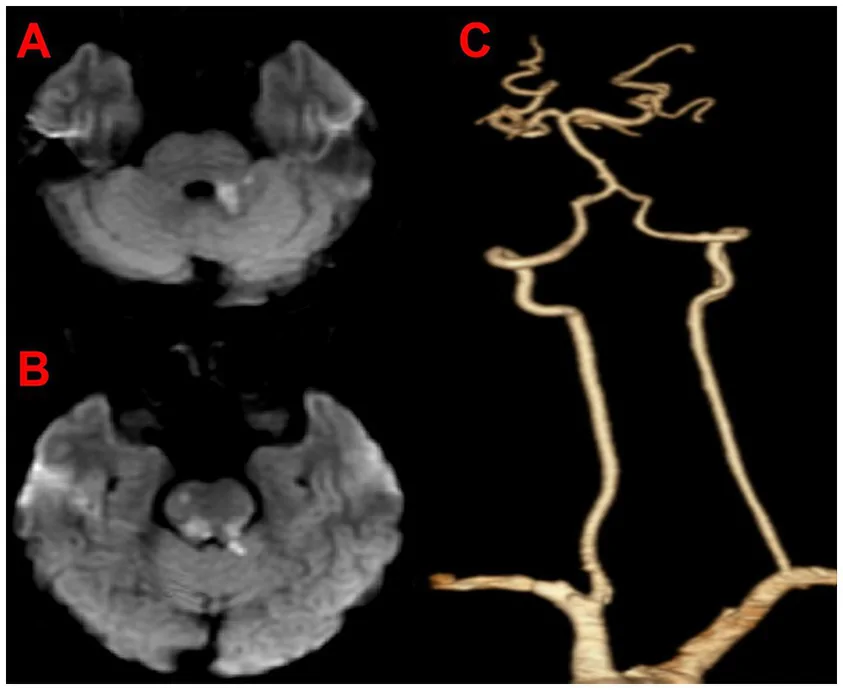
**(A,B)** DWI of the patient showed acute infarction in the brainstem and the left cerebellar hemisphere; **(C)** CTA of the head and neck showed no abnormalities.

**Figure 2:**
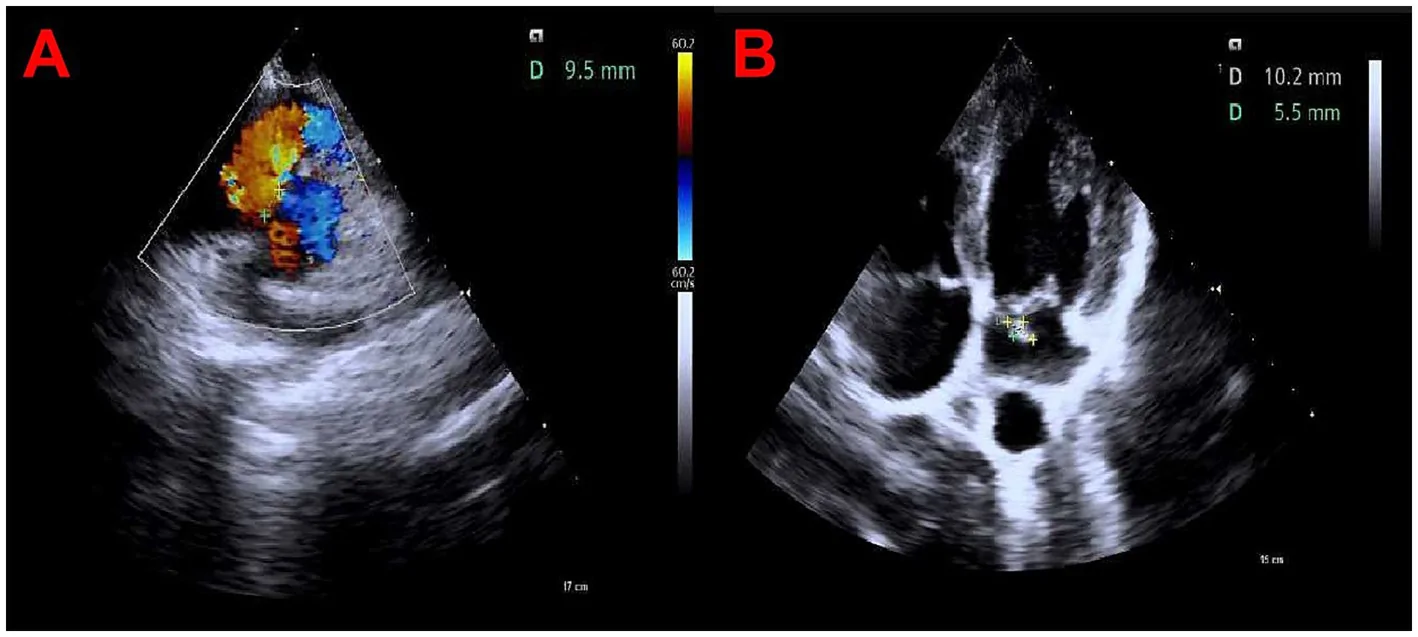
**(A)** Echocardiography of the patient showed a ventricular septal defect. **(B)** Echocardiography of the patient showed mitral valve vegetations.

**Figure 3:**
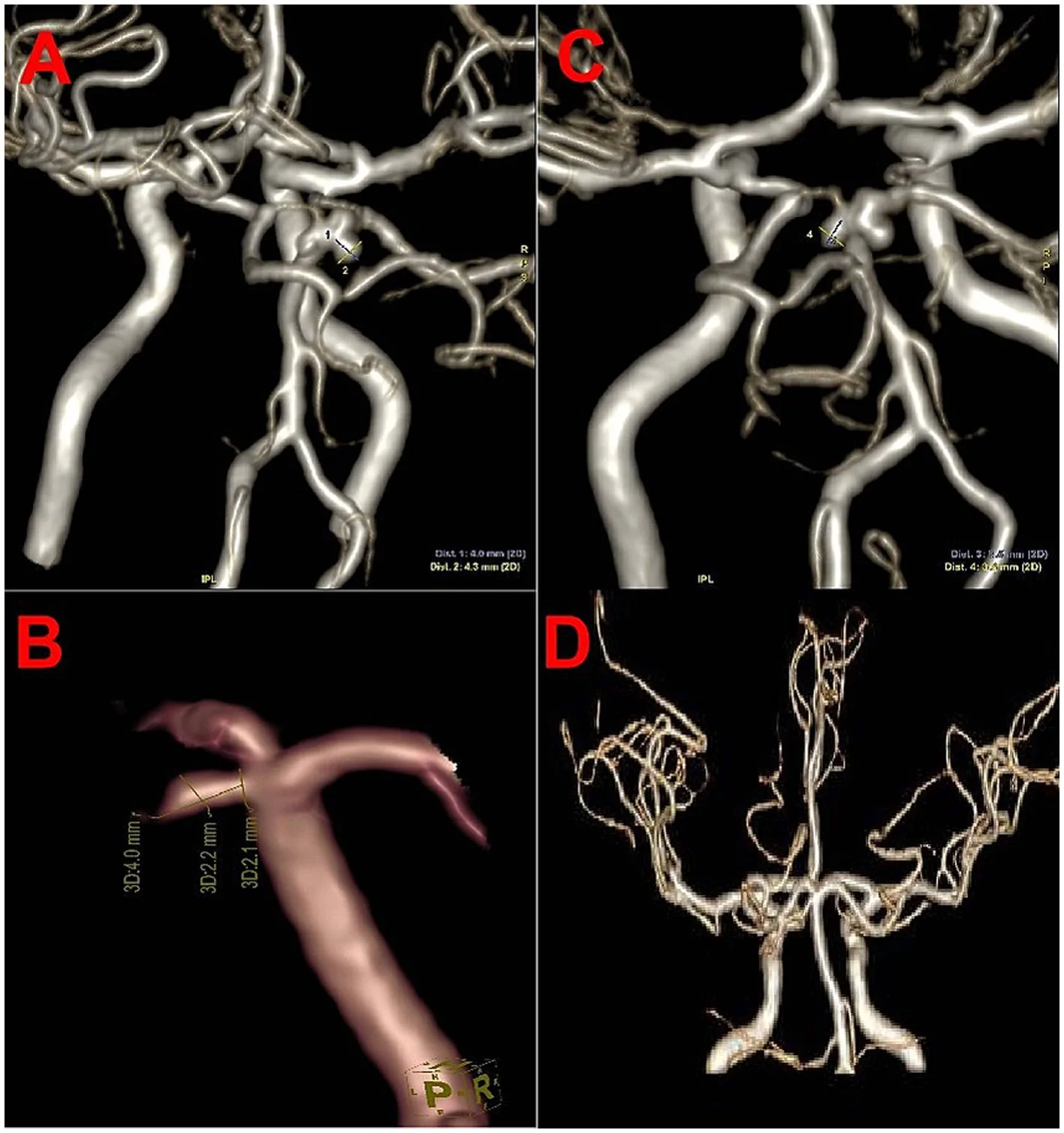
**(A,B)** MRA on the 10th day after admission showed multiple aneurysms in the basilar artery and stenosis of the P1 segment of the left posterior cerebral artery; **(C)** MRA on the 60th day after admission showed the left aneurysm of the basilar artery had enlarged, whereas the right aneurysm had disappeared, with persistent stenosis of the P1 segment of the left posterior cerebral artery; **(D)** MRA on the 278th day after admission showed complete recovery of the vascular lesions in the basilar artery and posterior cerebral artery.

## 3 Discussion

IIAs result from microbial infections and arterial wall degradation. While various pathogens can cause IIAs, bacterial infections predominate (72.8%), with *Streptococcus viridans* and *Staphylococcus aureus* being predominant causes, followed by fungal infections (13.2%) (5). Our case aligns with the literature in demonstrating that IE was complicated by sepsis and IBA formation with *Streptococcus viridans* bacteremia. However, this case has distinctive characteristics. While IIAs typically affect the middle and anterior cerebral arteries, our patient had basilar apex and posterior cerebral artery involvement with multiple aneurysms and concurrent stenosis—a rarely reported presentation.

The pathogenesis of IIAs involves the lodging of infected emboli at arterial bifurcations due to hemodynamic factors (8–10). Pathogens may penetrate the damaged endothelium or access the vessel wall via the vasa vasorum, initiating an inflammatory cascade that culminates in aneurysm formation (9–12). In our case, initial cerebral infarction suggested embolic occlusion of the basilar perforators, while subsequent aneurysm discovery highlighted the dynamic nature of this process. The transient enlargement of the aneurysm after anti-infective therapy may be explained as follows: Initial antibiotic therapy controls the pathogen but may also induce a more intense local inflammatory response, leading to temporary increases in the amount of necrotic tissue and in inflammatory edema. Under blood flow pressure, the aneurysm temporarily enlarges. With tissue repair, the vessel slowly absorbs necrotic and inflamed tissue, and scar tissue forms, contracting and sealing the aneurysmal cavity from within and from the periphery, which results in the disappearance of the aneurysm on imaging.

Current IIA management lacks standardized guidelines, which necessitates individualized approaches. Alawieh et al. proposed that, first, aneurysm management should be prioritized over cardiac surgery because of an anticoagulation-associated risk of rupture; then, medical management with close monitoring should be considered for unruptured aneurysms; and finally, intervention should be provided if medical management fails (5, 12). Observational studies have demonstrated that IIAs follow a dynamic clinical course, and prolonged anti-infective therapy prior to aneurysm detection is correlated with improved outcomes (7). In this case, the patient had received anti-infective therapy for 10 days before the aneurysm was discovered, and anti-infective treatment was continued for another 14 days. The patient’s inflammatory markers subsequently returned to normal, and fever resolved. Vascular follow-up imaging revealed that the aneurysm underwent several morphological changes and eventually disappeared, which is consistent with the conclusions of this study. During the acute phase of the disease, because cardiac surgery was not performed, anticoagulant therapy was administered to prevent recurrent cerebral infarction by reducing the continued shedding of cardiac emboli. In accordance with Chinese guidelines, oral enteric-coated aspirin is recommended for the secondary prevention of acute ischemic stroke in patients with unruptured aneurysms (13). Some studies have suggested that aspirin may help prevent the growth and rupture of unruptured aneurysms (14, 15). Therefore, the patient was prescribed oral enteric-coated aspirin (100 mg once daily) after discharge. In this case, an intracranial infectious aneurysm was detected 10 days after disease onset, but continuous vascular imaging of the aneurysm was not performed for 7–14 days. At the 60-day follow-up, the aneurysm was found to have enlarged, but no interventional procedure was performed, as the patient and his family declined continuous vascular imaging assessment and surgical treatment because of financial constraints. Although this posed a risk of rupture and hemorrhage, subsequent follow-up showed a favorable outcome with aneurysm resolution. These are the limitations of this case.

## 4 Conclusion

In conclusion, although IIAs are rare, their potential morbidity warrants cerebrovascular imaging in patients with systemic infections and neurological symptoms. This case demonstrates that complete resolution of select unruptured IIAs can be achieved with pharmacological management alone, establishing conservative therapy as a viable alternative when intervention is contraindicated or declined.

## Data Availability

The raw data supporting the conclusions of this article will be made available by the authors, without undue reservation.
