# Correction: Case Report: Medical management of a rare infectious basilar artery aneurysm

**DOI:** 10.3389/fmed.2026.1938797

**Published:** 2026-08-06

**Authors:** Jie Wang, Yuhua Lv, Lihao Liu

**Affiliations:** Banan Hospital of Chongqing Medical University, Chongqing, China

**Keywords:** case report, cerebral infarction, infectious intracranial aneurysm, infective endocarditis, medical management

Affiliation “School for Cardiovascular Diseases, Faculty of Health, Medicine and Life Sciences, Maastricht University, Maastricht, Netherlands” was erroneously included in the paper. This affiliation has now been removed for author “Jie Wang”.

The original version of this article has been updated.

